# Hyperproliferation is the main driver of metabolomic changes in psoriasis lesional skin

**DOI:** 10.1038/s41598-020-59996-z

**Published:** 2020-02-20

**Authors:** Liis Pohla, Aigar Ottas, Bret Kaldvee, Kristi Abram, Ursel Soomets, Mihkel Zilmer, Paula Reemann, Viljar Jaks, Külli Kingo

**Affiliations:** 1 Department of Dermatology and Venereology, University of Tartu, Tartu, Estonia; 2 Dermatology Clinic, Tartu University Hospital, Tartu, Estonia; 3 Department of Biochemistry, Institute of Biomedicine and Translational Medicine, University of Tartu, Tartu, Estonia; 4 Centre of Excellence for Genomics and Translational Medicine, University of Tartu, Tartu, Estonia; 5 Department of Cell Biology, Institute of Molecular and Cell Biology, University of Tartu, Tartu, Estonia

**Keywords:** Biomarkers, Skin diseases

## Abstract

Systematic understanding of the metabolite signature of diseases may lead to a closer understanding of the disease pathogenesis and ultimately to the development of novel therapies and diagnostic tools. Here we compared for the first time the full metabolomic profiles of lesional and non-lesional skin biopsies obtained from plaque psoriasis patients and skin samples of healthy controls. Significant differences in the concentration levels of 29 metabolites were identified that provide several novel insights into the metabolic pathways of psoriatic lesions. The metabolomic profile of the lesional psoriatic skin is mainly characterized by hallmarks of increased cell proliferation. As no significant differences were identified between non-lesional skin and healthy controls we conclude that local inflammatory process that drives the increased cell proliferation is the main cause of the identified metabolomic shifts.

## Introduction

Psoriasis is a chronic inflammatory skin disease that affects 1–2% of the population and has a profound impact on the quality of life of those affected by this condition^1,2^. There are a number of comorbidities associated with psoriasis: metabolic syndrome and its components (central obesity, atherogenic dyslipidemia, systemic arterial hypertension, insulin resistance), cardiovascular disease (CVD) and non-alcoholic steatohepatitis^3,4^.

The onset of psoriasis involves the interaction of genetical predispositions and environmental factors, however, the exact cause of the disease remains unknown. Experimental and clinical evidence point to the decisive role of immune system in the pathogenesis of the disease. Th-1 and Th-17 inflammatory cytokines are elevated in the skin and blood of psoriasis patients. In addition to promoting T-cell recruitment, angiogenesis and epidermal hyperproliferation, they also have a pleiotropic effect on adipogenesis, lipid metabolism and insulin signaling. On the other hand, the production of inflammatory cytokines that may modulate the development and severity of psoriasis is increased in the organism of patients suffering from obesity, atherosclerosis, thrombosis and diabetes thus creating a potentially vicious cycle sustaining the disease and facilitating its progression^5^.

Several metabolomic analyses of blood isolated from psoriasis patients have been published to date. Elevated levels of Glu, Phe, isoleucine (Ile), proline (Pro), ornithine (Orn), sulfoxidized methionine, ADMA, homocysteine (Hcy), various phosphatidylcholines, alpha ketoglutaric acid, lactic acid and urea were found in the blood of psoriasis patients when compared to the samples collected from healthy persons. Higher concentrations of choline, taurine, Glu, Phe, arachidonic acid, citrulline and 12-hydroxyeicosatetraenoic acid (12-HETE) and increased ratios of Glu to serine (Ser), creatinine to glycine (Gly) and taurine to Ala were found in psoriatic lesional skin compared to non-lesional skin or controls^6–11^. Several studies that investigated the levels of specific metabolite classes have been conducted^8,9,12,13^, however, to our best knowledge systematic metabolomic analysis of whole skin biopsies have not been performed yet.

In the current study we aimed to outline the metabolomic signature of psoriasis lesions and compared the metabolomic profiles of psoriasis patients lesional skin to that of non-lesional skin and healthy controls.

## Materials and Methods

### Ethics approval

This study was approved by the Research Ethics Committee of the University of Tartu. Permission number 245/M-18. The Declaration of Helsinki protocols were followed and patients gave their informed, written consent.

### Volunteer recruitment

The adult patients with plaque psoriasis were recruited from the Tartu University Hospital at the Clinic of Dermatology between 2013–2015. Controls were recruited either from the Clinic of Traumatology and Orthopaedics or from the Clinic of Dermatology. The exclusion criteria for participants were any other comorbid skin disease. 20 patients with plaque psoriasis (7 women, 13 men, ages 20–75) and 19 healthy controls (6 women, 13 men, ages 23–75) were enrolled to the study. Five of the psoriasis patients had psoriatic joint involvement and 7 had nail involvement. The comorbidities the psoriasis patients had were following: food allergies (1 patient), drug allergies (1), dust mite allergies (2), urticaria (no active rash at the time of biopsy) (2), diabetes (2), thyroid disease (1), rheumatoid arthritis (1) and hypertension (4). Nine of the 20 patients had positive family history of psoriasis. All of the participants were Caucasians of Eastern European descent and all participants provided written informed consent.

### Skin biopsies

3 mm punch biopsies were taken from the well-defined psoriatic lesional skin and adjacent (1–2 cm from lesions) visually non-lesional skin from upper arm and torso of psoriasis patients as well as similar locations that were not exposed to the sun in controls. The biopsies were collected before the first meal of the day. The skin samples were frozen immediately in liquid nitrogen and stored at −80 °C until needed. The samples were collected over a period of 3 years after which the metabolites were extracted and the samples were lyophilized. The lyophilized samples were stored at −80 °C until analysis as described before^14^. Prior to measurements the skin samples were weighed and a mix of 12 ml/g methanol and chloroform and 6 ml/g water was added according to the skin sample weight. 12 mm steel balls were added to the tube and milled using BulletBlender (NextAdvance). The sample was incubated for 1 h on ice, the supernatant was transferred to a clean tube and centrifuged at 16000 × g, and 4 °C for 15 minutes. The methanol/water and chloroform phases were pipetted to separate tubes and lyophilized.

### Metabolomic analysis

Absolute*IDQ* p180 kit (Biocrates Life Sciences AG, Innsbruck, Austria) was used for the targeted analysis of 188 metabolites. An Agilent Zorbax Eclipse XDB C18, 3.0 × 100 mm, 3.5 µm with Pre-Column SecurityGuard, Phenomenex, C18, 4 × 3 mm was used on a 1260 series HPLC (Agilent, USA) in tandem with a QTRAP 4500 (ABSciex, USA) mass-spectrometer. The exact protocol is detailed in the user’s manual of the *AbsoluteIDQ p180* kit. Briefly, the lyophilized samples were thawed on ice, the two lyophilized phases were both dissolved in 85% methanol/ 15% water according to their previous weight (15–25 μl of added solvent) and both phases were added to the filter plate of the kit. Subsequently 10 µl of internal standards were added. The samples were derivatized using phenylisothiocyanate, dried and metabolites extracted using 40% methanol in water. Acetonitrile, chloroform, formic acid (FA), methanol and water were all HPLC grade and purchased from Sigma-Aldrich (Germany).

### Data analysis

Data was analyzed using R version 3.5.1^15^. The non-parametric Kruskal-Wallis rank-sum test and Wilcoxon rank-sum test were used when looking for phenotype differentiating metabolites. Probabilities were adjusted using FDR 5% and the corrected p-value < 0.05 was considered as statistically significant.

## Results and Discussion

The knowledge about the alterations in the metabolome of psoriasis patients is still modest and to a large extent based on data obtained from serum or plasma samples. There are only a few metabolomic studies that have analyzed skin samples^7–9^. To systematically identify the shifts in metabolomics profile of the psoriasis lesions we compared the metabolomic profiles of lesional (PS-L) skin to non-lesional (PS-NL) skin pairwise obtained from psoriasis patients and to control skin samples obtained from healthy controls (C).

**Figure 1:**
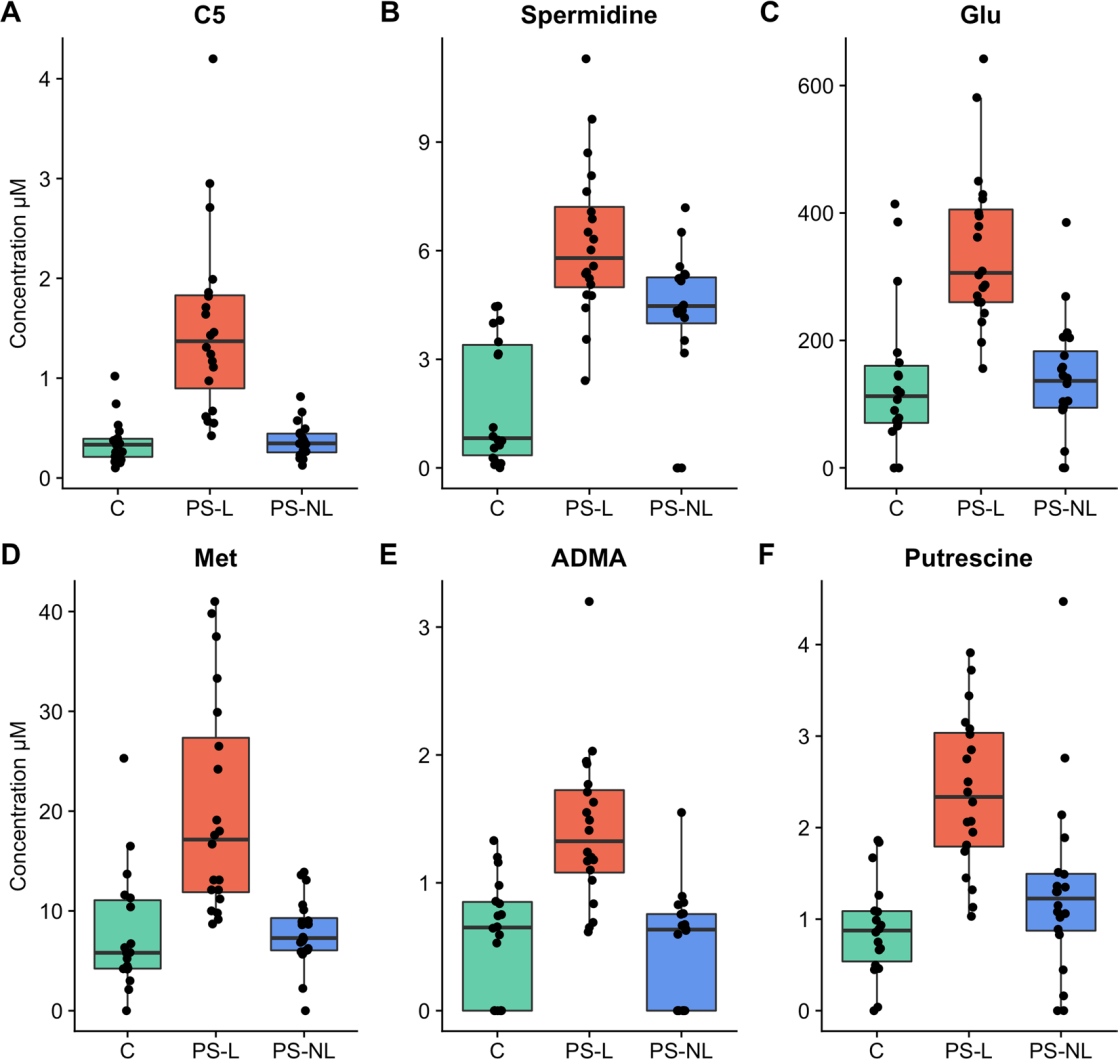
Boxplots of concentrations of six metabolites which concentrations were statistically significantly (Kruskal-Wallis rank sum test, FDR 5% corrected p-value < 0.05) elevated in psoriatic lesional skin (PS-L) compared to psoriatic non-lesional skin (PS-NL) and the skin of healthy controls (C). The levels are reported in micromolar (μM) concentrations. C5, valeryl-L-carnitine; Spermidine; Glu, glutamate; Met, methionine; ADMA, asymmetric dimethylarginine; Putrescine.

**Figure 2:**
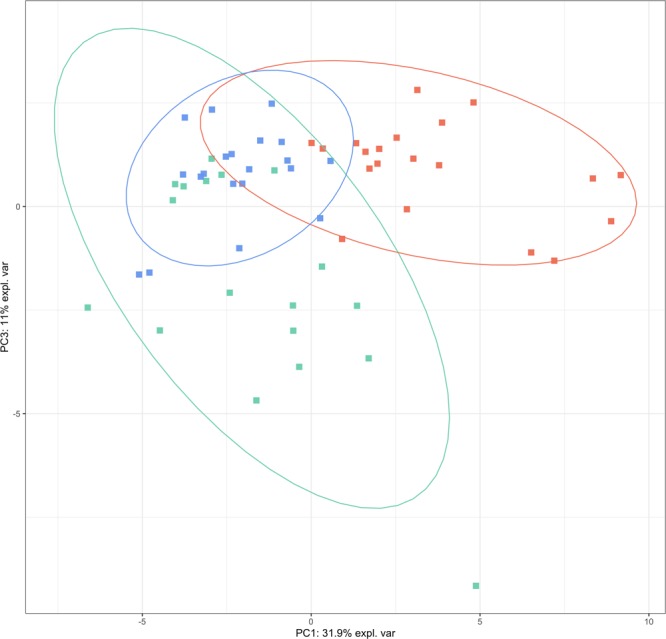
PCA plot of the targeted analysis. Psoriatic lesional skin samples are marked as red squares, psoriatic non-lesional skin samples are marked as blue triangles and control skin samples are marked as green circles. X and Y axes represent the percentage of variability explained by principal components one and two. Data is based on statistically significantly changed metabolites from Kruskal-Wallis test.

We found remarkable shifts in the metabolomic signature of psoriatic lesions as the concentration of 29 metabolites differed significantly in PS-L samples when all three sample groups were analysed (Kruskal-Wallis rank sum test, FDR 5% corrected p-value < 0.05; Table 1). The concentrations of 17 metabolites that included amino acids (AAs), acylcarnitines, biogenic amines, lysophosphatidylcholines, phosphatidylcholines, histamine and ADMA were increased and 2 metabolite ratios – citrulline to ornithine and ornithine to arginine – were decreased in PS-L skin compared to C (Fig. 1). The ratio of citrulline to ornithine was also decreased in PS-L skin compared to PS-NL skin. There were 14 metabolites, which concentration levels were elevated in PS-L skin compared to both C and PS-NL skin and 9 metabolites which concentrations were increased only in PS-L skin compared to PS-NL skin. Interestingly, no statistically significant differences between the metabolite profiles of PS-NL and C were found (data not shown). In general our findings showed that the metabolomic profile of PS-L skin is clearly different from that of C and PS-NL skin in principal component analysis of statistically significantly changed metabolites (Fig. 2). Interestingly, we did not find statistically significant correlations between psoriasis area severity index (PASI) and the measured metabolites in the lesional skin of psoriasis patients (Spearman’s correlation, FDR 5% correction for p-values).

**Table 1:** Statistically significantly different metabolites and their ratios between samples obtained from plaque psoriasis lesional (PS-L) and non-lesional (PS-NL) skin and the skin of healthy controls. Values are given in micromolar (μM) concentrations.

Metabolite	Healthy controls mean (µM)	PS-L mean (µM)	PS-NL mean (µM)	KW p-value FDR 5% corrected
**Amino acids**				
Glu	139.444	342.85	139.52	0.0001
Met	7.828	20.142	7.852	0.0002
Arg	49.728	136.455	60.55	0.0042
Lys	50.179	83.16	38.261	0.0063
Leu	50.924	90.3	31.96	0.0079
Met.SO	1.559	1.255	0.435	0.0323
Phe	31.434	49.705	22.78	0.0329
**Dimethylarginines**				
ADMA	0.571	1.419	0.475	0.0002
total.DMA	0.068	0.519	0.034	0.0002
**Biogenic amines**				
Spermidine	1.782	6.234	4.178	< 0.0001
Putrescine	0.889	2.383	1.31	0.0002
Histamine				
Histamine	11.195	21.197	14.976	0.0138
**Carnitines**				
C5	0.359	1.52	0.372	< 0.0001
C2	3.636	4.32	3.041	0.0063
C18.2	0.026	0.041	0.025	0.0063
C16	0.066	0.096	0.061	0.02
C18.1	0.059	0.075	0.044	0.0207
C18	1.589	3.265	1.93	0.0368
**Metabolite ratios**				
Cit…Orn	4.914	2.526	8.168	0.0018
Orn…Arg	0.763	0.193	0.378	0.0048
Fisher.ratio	1.847	2.025	1.698	0.0237
X.C2.C3….C0	0.35	0.306	0.289	0.0339
**Glycerophospholipids**				
PC.ae.C36.0	0.364	1.643	0.358	0.0003
lysoPC.a.C16.1	0.585	0.76	0.335	0.0064
PC.ae.C38.0	0.196	0.392	0.189	0.0125
PC.ae.C34.0	0.402	0.85	0.4	0.0244
PC.aa.C42.4	0.024	0.038	0.02	0.0248
PC.ae.C38.1	0.177	0.423	0.219	0.0323
PC.aa.C40.4	0.233	0.37	0.239	0.0386

In the amino acid group we identified significantly higher concentrations of Glu (p = 0.0020), Met (p = 0.0027) and Arg (p = 0.0049) in PS-L skin when compared to C skin. Similarly, the levels of Glu (p = 0.0003), Met (p = 0.0004), Arg (p = 0.0107), Lys (p = 0.0017), Leu (p = 0.0008) and Phe (p = 0.0114) were higher in PS-L skin when compared to PS-NL skin.

Our results are in concert with previous studies that showed increased Glu and Phe levels in lesional skin^7^. In addition, we also found increased Lys levels in PS-L skin. The increase in Glu concentration is potentially driven by the increased demand for glutamine during keratinocyte hyperproliferation, rapid protein synthesis and enhanced immune cells activity that are characteristic to PS^6,16,17^. Chronic inflammation causes the reduction of Phe conversion to Tyr and, consequently, higher Phe levels could be found in tissues with chronic inflammation^18^. Lys is related to collagen production, which is increased in psoriasis skin^19^. In addition Lys is required for biosynthesis of L-carnitine (Car) that was also increased in psoriasis lesions (our data and^20^). The increase in Glu and Phe levels are well corroborated by the previously found increase of these AAs in the blood of psoriasis patients^21^.

The Fisher ratio – the molar ratio of BCAA (Leu, Val, Ile) to aromatic amino acids (Phe, Tyr) – was significantly higher in PS-L skin compared to PS-NL skin (p = 0.01). The cachexic metabolic load in psoriasis may intensify the catabolism of glycoketogenic BCAA (Val, Ile, Leu) yielding both in elevation of Glu levels and short-chain acylcarnitines (SCACs) like C3 and C5 (derived from Val, Ile). Accordingly, an elevation of C5 in PS-L skin was found in this study (see below). Among other tasks, the metabolism of BCAAs is involved in immunity as these are required for lymphocyte proliferation and participate in dendritic cell maturation^22^.

As noted above, we found elevated levels of Met in PS-L skin that could oxidize to methionine sulfoxide (Met.SO) and therefore functions as a marker for oxidative stress^23^. Ottas *et al*. found that Met.SO level and Met.SO ratio to Met were significantly higher in psoriasis patients blood compared to controls^21^. In this work we found no significant differences in Met.SO levels between PS-L and C skin samples suggesting that there is no excessive protein-related oxidative stress in psoriatic skin lesions. This conclusion is supported by the presence of the increased levels of polyamines in PS-L skin as these are anticipated to provide protection from oxidative damage.

Three amino acids citrulline, ornithine (Orn) and Arg are metabolically tightly interrelated. The metabolism of citrulline is divided into two branches: the production of citrulline and the citrullination of proteins. Citrulline production from Arg involves three key enzymes: NO synthase, Orn carbamoyltransferase as well as argininosuccinate synthetase that further converts citrulline into argininosuccinate. Protein citrullination occurs as the result of post-translational modification (deimination) of Arg. Citrulline-containing proteins originate mainly from keratin K1. These are located in the cornified layers of epidermis and in psoriatic lesional skin the levels of citrullinated proteins are undetectable or markedly decreased^24,25^. Concordantly, alterations in urea cycle present in psoriasis lead to accumulation of intermediate metabolites and decrease citrulline levels^7,11,26,27^. Furthermore, the level of citrulline in PS lesions is negatively correlated with the plaque severity scores^7^.

Contrary to the notion that Arginase 1, which converts Arg to Orn and urea, is overexpressed in lesional skin^28^, we found the levels of Arg significantly increased in PS-L skin compared to PS-NL skin and C (p = 0.0107). In parallel, the ratio of Orn to Arg in lesional skin was decreased compared to controls suggesting lower arginase activity in PS lesions. The ratio of citrulline to Orn was also lower in PS-L skin compared to PS-NL skin and C being consistent to the lower activity of ornithine carbamoyltransferase found previously in the blood of psoriasis patients^21^. Of note, the levels of citrulline and Orn did not differ significantly between the three groups.

Arg metabolism related ADMA level was 2.5 times higher in PS-L skin compared to C (p = 0.0027) and 3 times higher compared to PS-NL skin (p = 0.0004). In line with previous findings the analytical sum of ADMA and SDMA (total DMA level) was 7.7 times higher in PS-L skin compared to C (p = 0.0052) and 15.4 times higher compared to PS-NL skin (p = 0.0005). The synthesis of ADMA is closely connected to Arg and in line with our data it has been previously found that psoriasis patients have higher ADMA serum levels than controls^29,30^.

The concentration of a diamine putrescine was 2.7 times higher in PS-L skin compared to C (p = 0.00001) and 1.8 times higher than in PS-NL skin (p = 0.0054). The concentration of polyamine – spermidine – was 3.5 times higher in PS-L skin compared to C (p = 0.000002) and 1.5 times higher than in PS-NL skin (p = 0.03). Additionally, there were no significant differences in spermidine and putrescine levels between PS-NL skin and C. Spermidine and spermine are two major polyamines produced in all mammalian cells that are produced from putrescine via decarboxylation of Orn^31^. Polyamines contribute to cell growth, proliferation, differentiation, gene regulation, protein and nucleic acid synthesis. They are synthesized intensively during cell division and provide protection from oxidative damage as well as contribute to nucleic acid structure and stability^31–34^. In line with previously published data we found that the levels of spermidine and putrescine were higher in PS-L skin compared to the PS-NL skin and C being consistent with the presence of excessive cell proliferation in the psoriatic lesions^33^.

The concentration of histamine was also significantly elevated in PS-L skin when compared to C (p = 0.0072). It has been found that 65–80% of the patients with PS complain itching and histamine is an important mediator of this symptom^35–37^. Consistent with our results, it has been found that histamine levels in psoriatic plaques are significantly higher than in controls^37^.

We also found the differences in 6 acylcarnitines (C2, C5, C16, C18, C18.1, C18.2). The levels of octadecanoyl-L-carnitine (C18) were significantly higher in PS-L skin compared to C (p = 0.0347). The concentrations of valeryl-L-carnitine (C5), hexadecanoyl-L-carnitine (C16) and octadecadienyl-L-carnitine (C18.2) were higher in PS-L skin compared both to PS-NL skin and C. In addition, the concentrations of acetyl-L-carnitine (C2) and octadecenoyl-L-carnitine (C18.1) were higher in PS-L skin compared to PS-NL skin.

Carnitine (Car) is an essential participant in fatty acid metabolism as it transports the fatty acids into the mitochondrial matrix to support energy metabolism through followed fatty acid β-oxidation. In addition to Lys, the concentration of Met was increased in PS-L skin suggesting the presence of an activated protein turnover and acting as a prerequisite for the increase in Car synthesis in the psoriatic lesions. As described above, the endogenous Car pool consists of Car and various short-, medium- and long-chain acylcarnitines (SCACs, MCACs and LCACs, respectively). In PS-L skin the concentrations of LCACs were elevated. MCACs and LCACs may also have a pro-inflammatory role and trigger cellular stress that is well concordant with the inflammatory status of the psoriatic plaques^38^.

Interestingly, as noted above, we found an elevated level of C2 (acetylcarnitine, ALCAR) in PS-L skin. ALCAR is an important marker of cell energetic and metabolic homeostasis, more particularly, the level of coenzyme A (CoA). One of the means to regulate level of acetyl-CoA (AcCoA) is by transferring the acetyl moiety from AcCoA to Car that is thereby converted into ALCAR. Thus, the production of ALCAR elevates free pool of coenzyme A that could be further utilized for improvement of glucose metabolism via enhancing the efficiency of pyruvate dehydrogenase, synthesis of very long chain fatty acids and protein acetylation. Therefore, elevated level of ALCAR points to the modulation of the intracellular coenzyme A homeostasis reflecting thereby the metabolic status of the skin cells^39^.

There were 7 glycerophospholipids (PC.ae.C36.0, lysoPC.a.C16.1, PC.ae.C38.0, PC.ae.C34.0, PC.aa.C42.4, PC.ae.C38.1, PC.aa.C40.4; a – acyl, aa – diacyl, ae – acyl-alkyl) that differed significantly between the experiment groups. The concentrations of phosphatidylcholines PC.ae.C36.0, PC.ae.C34.0 and PC.ae.C38.1 were higher in PS-L skin compared both to PS-NL and C. The level of PC.aa.C42.4 was 1.9 times higher in PS-L skin compared to PS-NL skin (p = 0.0196) and the level of PC.aa.C40.4 was 1.5 times higher in PS-L skin compared to C (p = 0.0439). The concentration of another lipid, lysophosphatidylcholine acyl C16:1 (lysoPC.a.C16.1) was also significantly higher in PS-L skin compared to PS-NL skin (p = 0.0017). The data is in good accordance with the increased cell proliferation present in the psoriatic plaques as PC-s are essential constituents of cell membranes. Furthermore, it has been found that lysophosphatidylcholines induce T-lymphocyte chemotaxis and thereby help to maintain the chronic inflammation found in psoriatic epidermis^40^.

Although it is well known that proliferating cells require more glucose for growth and consequently the psoriatic keratinocytes are anticipated to take up more glucose we found no significant differences in the hexose group of metabolites. A plausible explanation would be that the increase in glucose consumption is readily compensated by the increased blood supply present in psoriatic plaques.

In conclusion, we compared the metabolomic profiles of the lesional and non-lesional skin of patients suffering from plaque psoriasis and the skin of healthy controls. While our data is in agreement with previous findings we provide several novel insights into the metabolic environment of psoriatic lesions. The metabolomic profile of the lesional psoriatic skin is mainly characterized by hallmarks of increased cell proliferation when compared to non-lesional and control skin. As there were no significant differences between the latter two sample groups one has to conclude that the local inflammatory process that drives the cell proliferation is the main cause of the identified metabolomic shifts.
